# Comparative evaluation of Chloroquick with Triphala, sodium hypochlorite, and ethylenediaminetetraacetic acid on the microhardness of root canal dentin: An *in vitro* study

**Published:** 2021-01-21

**Authors:** Vaishnavi Elika, Divya Kunam, Lavanya Anumula, Suneel kumar Chinni, Kiranmayi Govula

**Affiliations:** Conservative Dentistry and Endodontics, Narayana Dental College and Hospital. Chintareddypalem, Nellore, India

**Keywords:** Chloroquick, Triphala, microhardness, etidronic acid, apical third, radicular dentinal surface

## Abstract

**Background:**

Irrigating solutions used for the elimination of microorganisms during root canal preparation affect the chemical and physical properties of dentin, thereby rendering the tooth more prone to fracture. Therefore, the careful and judicious selection of irrigant is required which has maximum benefits with minimum undesirable properties.

**Aim:**

The study aimed to compare and evaluate the effect of Chloroquick with composition of 18% etidronic acid+ 5% sodium hypochlorite (NaOCl) with other irrigants such as Triphala, NaOCl, and ethylenediaminetetraacetic acid (EDTA) on the microhardness of root canal dentin.

**Methods:**

Forty freshly extracted non-carious single-rooted human teeth were collected and decoronated at CEJ to standardize the canal length. The roots were sectioned longitudinally to get two halves. Baseline microhardness evaluation was done using Vickers microhardness test before the immersion in irrigants; samples were then randomly divided into four groups (*n*=20), based on the irrigant used as follows: Group 1 – Saline; Group 2 – 5% NaOCl +17% EDTA; Group 3 – Triphala; and Group 4 – Chloroquick. Later, the samples were immersed in the irrigating solutions for 15 min at 37°C for each group and were then subjected to post-treatment microhardness testing. Microhardness values were recorded and statistically analyzed using one-way ANOVA and intergroup comparison with *post hoc* Tukey test (*P*<0.05).

**Results:**

The results of the present study showed that all the tested specimens showed a decrease in the microhardness values following application of different irrigating solutions except the control group. The use of Triphala and Chloroquick has minimal effect on the microhardness of root canal dentin post-treatment when compared with 5% NaOCl and 17% EDTA.

**Conclusion:**

Chloroquick, as well as 0.005% Triphala, can be used safely as an irrigating solution with less detrimental effects on the hardness of root dentin.

**Relevance for Patients:**

The newer irrigant Chloroquick shows less effect on dentin microhardness, thereby reducing the incidence of root fractures in patients postoperatively.

## 1. Introduction

Root canal disinfection by instrumentation and irrigation is considered as the most important factor in the prevention and treatment of endodontic diseases [1]. The previous studies have reported that endodontic irrigation is able to cause alterations in the chemical composition of dentin. These changes arise because of the variations in the inorganic and organic phase of dentin, thereby decreasing the microhardness of root canal dentin rendering the tooth more prone to fracture. Therefore, the careful and judicious selection of irrigant is required, which has maximum benefits with minimum undesirable properties [2]. Sodium hypochlorite (NaOCl) in 1–5.25% concentration is the most widely recommended irrigation solution because of its tissue dissolving and antimicrobial capability [3]. In a study, the use of 17% ethylenediaminetetraacetic acid (EDTA) and 5% NaOCl as an irrigating solution individually and NaOCl followed by EDTA in alteration stated that all the irrigating solutions except for distilled water significantly decreased dentin microhardness. This is due to the strong chelating property of EDTA [4].

There has been a growing trend to seek natural remedies as a part of dental treatments. Herbal or natural products have been used in dental and medical practice for thousands of years. They are becoming even more popular today, due to their high antimicrobial activity, biocompatibility, anti-inflammatory, and antioxidant properties [5]. Triphala is an Ayurvedic herbal formulation comprised dried, powdered fruits of three medicinal plants *Terminalia bellirica*, *chebula* and *Emblica officinalis*. Tannic acid is the principal constituent of Triphala [6]. Initial studies have shown that tannic acid has bacteriostatic and bactericidal activity against Gram-positive and Gram-negative bacteria [7]. Studies by Shakouie *et al*. and Prabhakar *et al*. have concluded that Triphala has better antibacterial activity than 0.5% and 1% NaOCl [8,9]. It has many applications in dentistry as an endodontic irrigant [9], mouth wash [10], matrix metalloproteinase inhibitor in periodontal diseases [11], and its anti-caries activity have also been reported [12]. An *in vitr*o study by Asghari *et al*. stated that Triphala has the least effect on microhardness of root canal dentin when compared to 5.25% NaOCl and 2% chlorhexidine [13].

Recently, etidronate solution (HEBP) emerged as a substitute for the commonly used chelators. It is a weak chelator that appears to have a nominal effect on the dentinal walls and hence shows a less aggressive effect than EDTA on the root canal dentin [14]. A newer formulation Chloroquick (Innovationsendo, India) which is a one-step irrigating solution containing 18% etidronic acid and 5% NaOCl has been introduced as an alternative to EDTA [15]. Chloroquick solution is available in the form of two vial or bottle systems. Chloroquick high contains 5% NaOCl and 18% etidronic acid, which acts as an activator whereas Chloroquick low contains 3 % NaOCl and 9% etidronic acid. Both are to be mixed freshly according to the manufacturer’s instructions for a complete root canal irrigating solution. In the current study, Chloroquick high was used. The aim of this *in vitro* study was to evaluate and compare the microhardness of root canal dentin using Chloroquick with respective irrigants in the apical third of the root canal dentin using Vickers microhardness testing machine.

## 2. Materials and Methods

### 2.1. Preparation of Triphala powder solution

0.5 g of commercially available Triphala powder (IMPCOPS Ltd., Chennai, India) was weighed and dissolved in 1000 ml dimethyl sulfoxide (DMSO) (SD Fine Chemicals,

Chennai, India) to obtain an irrigating solution with a concentration of 5 mg/ml. DMSO was used as a solvent for Triphala, although it is readily soluble in water. DMSO is a clean, safe, highly polar solvent that helps in bringing out pure properties of the herbs being dissolved in it [16]. The solution was stirred using a mechanical stirrer to remove the impurities for about 120 min to obtain a freshly concentrated irrigating solution.

5% NaOCl and 17% EDTA (SS traders, India), and Chloroquick high contains 5% NaOCl and 18% etidronic acid (Innovationsendo, India) were used in the study.

### 2.2. Specimen preparation

**Figure 1:**
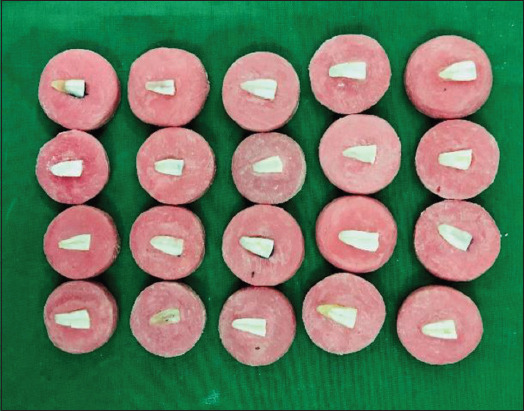
Samples mounted in acrylic resin blocks.

The study protocol was approved by the Institutional Ethical Committee (IECC NDCH/2019/P-07). Forty single-rooted freshly extracted human teeth without caries, fractures, and unrestored are collected and were stored in 0.1% thymol solution. The samples were decoronated at the level of the cementoenamel junction with the help of a diamond impregnated disc to standardize the canal length. No endodontic treatment was initiated for the samples. The roots were sectioned longitudinally by placing grooves on the buccal and lingual external surface of the roots to make them into two halves using a double-faced diamond disc under water cooling. Eighty dentin specimens thus obtained were then ground polished using a fine gritted silicon carbide abrasive paper (600 grit and 1200 grit). The freshly mixed autopolymerized resin was poured in plastic rings of uniform diameter. All the samples were embedded in acrylic resin blocks of uniform size 2×2 cm with polished side facing outwards (Figure 1). Later, repolishing of the specimen was done after mounting on the resin molds to remove excess material present on the tooth surface.

### 2.3. Baseline microhardness evaluation (T_0_)

**Figure 2:**
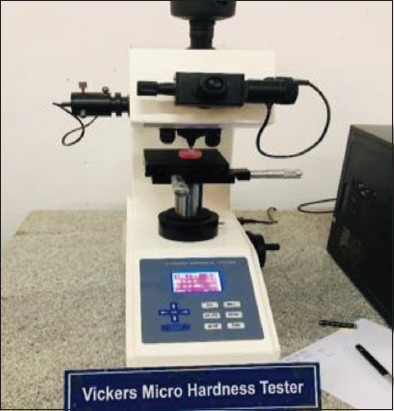
Microhardness evaluation using Vickers hardness machine.

Microhardness of all the samples was determined using a Vickers microhardness tester fitted with a 200 g load (Figure 2). The diamond indenter was allowed to sink on the root canal dentin surface for 20 s at the apical third, and the Vickers hardness number was determined. Three indentations were made on each specimen at a mean distance of 100 mm, and the representative hardness value for each specimen was obtained as the average measurement of the three indentations. This was taken as the baseline (T0) microhardness value of the sample.

**Figure 3:**
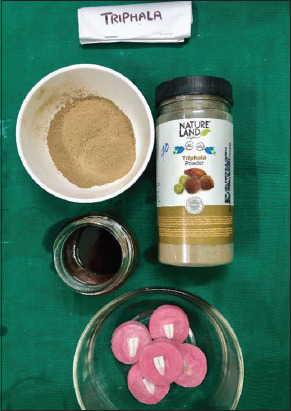
Samples treated with Triphala in closed glass plates.

**Figure 4:**
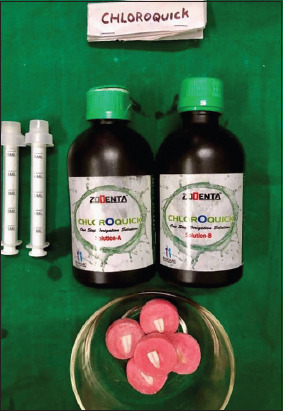
Samples treated with Chloroquick in closed glass plates.

Later, the samples were randomly divided into four groups. All the samples were immersed in the irrigating solutions with a mean time of 15 min. Saline was used as immersing solution for all the samples in Group 1. About 5% NaOCl and 17% EDTA was used alternatively with a mean time of 7.5 min each as an immersing solution for the samples in Group 2, and in a similar way, Triphala (Figure 3) and Chloroquick (Figure 4) were used for the samples in Group 3 and Group 4, respectively.

### 2. 4. Post-treatment microhardness evaluation (T_1_)

After immersion of the samples in the respective irrigating solutions, they were washed with distilled water to avoid prolonged contact with the test irrigating solution. The samples were dried thoroughly, and the microhardness of the samples was measured again. This value was taken as the post-treatment (T1) microhardness value of the sample.

### 2.5. Statistical analysis

Data analysis was carried out using the Statistical Package for the Social Sciences (SPSS version 21). Descriptive statistics were presented in the form of minimum, maximum, mean, and standard deviation (SD). Data were assessed using analysis of variance (ANOVA) to test the overall difference among baseline and post-treatment for microhardness. *Post hoc* Tukey test was used for intergroup comparisons. Paired *t*-test was used to determine significant difference between baseline and post-treatment values. The level of significance was set at *P*<0.05 for all tests.

## 3. Results

**Figure 5:**
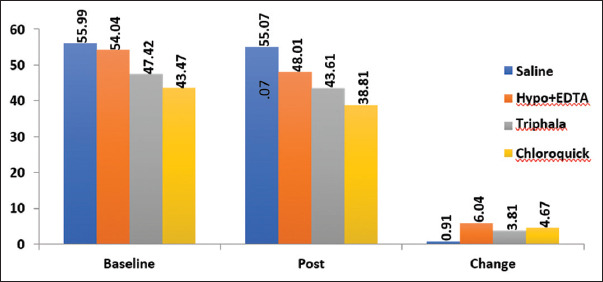
Post hoc comparison of microhardness values before and after treatment in each study groups at apical third with respect to type of treatment.

The baseline and post-treatment mean microhardness values, mean, and SD of all the groups are summarized in Table 1. The baseline microhardness values showed no significant difference among the groups (*P*>0.05), indicating uniform distribution of samples between the groups. All the irrigating solutions except Group 1 (control group) showed reduction in the microhardness. *Post hoc* comparison (Figure 5) showed percentage reduction in the Vickers microhardness values after the use of irrigating solutions in the following order Group 2 >Group 3 and Group 4 (*P*>0.05) >Group 1.

**Table 1:** Comparison of microhardness before and after treatment in each study group.

Groups	Frequency	Mean±S. D	*P* value
Group 1			
Pre-treatment	20	55.98±3.94	0.447**
Post-treatment	20	55.07±4.15	
Group 2			
Pre-treatment	20	54.03±5.88	<0.001*
Post-treatment	19	48.00±5.32	
Group 3			
Pre-treatment	20	47.40±5.53	<0.001*
Post-treatment	20	43.60±5.95	
Group 4			
Pre-treatment	20	43.46±4.43	<0.001*
Post-treatment	20	38.80±4.90	

Paired “*t*”-test,

* *P*<0.05 (significant),

** *P*>0.05 (not significant)

## 4. Discussion

The irrigating solutions might influence the physicochemical properties of human root canal dentin, including microhardness, permeability, roughness, and wettability. The microhardness determination gives indirect evidence of mineral loss or gain in tooth hard structures. The degree of mineral content and the amount of hydroxyapatite in the intertubular substance are helpful in determining the intrinsic hardness characteristics of dentin structure. Thus, the decrease of dentin microhardness probably contributes to an increase in the incidence of fractures or cracks [17]. Vickers hardness test is widely accepted and is considered as a highly suitable and practical method to evaluate the change in the surface in deeper hard tissue structures [18].

In this study, all the specimens were immersed in their respective irrigating solutions for 15 min before subjecting for microhardness testing. Goldberg *et al*. [19,20] have suggested the application time of 10–15 min to obtain optimal results, which is more realistic in terms of clinical practice. However, other researchers limited the contact time to 5 min [21]. The location of radicular dentin is also another determinant in affecting microhardness as there is an inverse correlation between tubule density and microhardness [22]. Hence, in the present study, the apical third portion of the dentin was evaluated in determining the dentin microhardness.

The previous studies [23] had shown that there was a decrease in microhardness of root canal dentin when 5.25% NaOCl, 17% EDTA, and chlorhexidine were used as the irrigating solutions. The reason for the decrease in the hardness of root dentin might be decrease in stiffness of the intertubular dentin matrix caused by the heterogeneous distribution of the mineral phase within the collagen matrix [24]. Apart from this, the microhardness of root dentin is dependent on the concentration of NaOCl employed. The concentration of NaOCl has inverse effects on the elastic modulus and flexural strength [25]. EDTA, due to its chelating property, induces an adverse softening potential on the calcified portion of dentin. The reduction in dentin microhardness was expected as its entire cationic receptors are saturated with calcium ions of root dentin [4]. In the present study, the results of post-treatment microhardness values of 5% NaOCl and 17% EDTA have shown no exception with the published data which show that there was a decrease in the microhardness values post-treatment.

Triphala has shown less reduction in the microhardness of root dentin when compared to 5% NaOCl and 17% EDTA. A study by Mahsa *et al*. [13] concluded that microhardness of root canal dentin had a minimal effect when Triphala was used as an irrigating solution. The most probable reason for this might be because of the citrus acid present in the fruits of Triphala, which acts as a weak chelator.

In the field of endodontics, Chloroquick high was evaluated for the smear layer removal from the root canal dentin and concluded that it was able to remove the smear layer as effectively as conventional irrigating solutions [26]. This study has focused on the evaluation of microhardness of root canal dentin using synthetic as well as herbal irrigants. It is evident from our study that the percentage reduction of microhardness was less with Triphala, but Chloroquick showed less reduction in post-treatment microhardness of root dentin than 5% NaOCl and 17% EDTA and the results was in accordance with the recent study by Pritee *et al*. [27]. The reason might be due to etidronate; an activator in Chloroquick high solution that acts as a weak chelating agent and attacks less dentin surface and forms complexes with calcium ions compared to other commonly used irrigants such as EDTA or citric acid. However, further clinical studies are required regarding its safety and biocompatibility.

## 5. Conclusion

Within the limitations of this *in vitro* study, it can be concluded that

- All the used irrigants affected the microhardness of human radicular dentin.
- Chloroquick has less detrimental effects on the microhardness of root dentin compared to NaOCl and EDTA.
- 0.005% Triphala and Chloroquick are equally effective as irrigating solutions.
